# Ex ante economic impact assessment of the 3R-gene potato in Kenya

**DOI:** 10.1371/journal.pone.0309329

**Published:** 2025-03-31

**Authors:** Evelyne Kihiu, Marc Ghislain, Anthony Mwangi Kibe, Ng’ang’a Nancy, Marcel Gatto, Jose B. Falck-Zepeda

**Affiliations:** 1 International Potato Center (CIP), Nairobi, Kenya; 2 Department of Crops, Horticulture and Soil, Egerton University, Njoro, Kenya; 3 Kenya Agricultural Livestock and Research Organization-Tigoni, Limuru, Kenya; 4 International Potato Center, Hanoi, Vietnam; 5 International Food Policy Research Institute (IFPRI), Washington, D.C., United States of America; ICAR - Indian Agricultural Research Institute, INDIA

## Abstract

Potato late-blight disease is as a major constraint to potato production in Kenya. The use of fungicides to control the disease is limited by the practice of delaying application until the symptoms are visible, and inappropriate application rates and methods. Biotech crops, such as 3R-gene potato, are providing sustainable solutions to crop protection challenges in agriculture, but little is known about their social and economic potential in any country. To gain greater insights into the potential of 3R-gene potato in Kenya, this study evaluates the economic benefits of Asante, Shangi, and Tigoni potato varieties, which are resistant to late blight. Data from experts along the potato value chain and secondary sources are analyzed using the economic surplus model and a real options model. First, experts’ opinions revealed that late-blight disease is responsible for 23% of production loss annually, and that 12% of the production costs are due to the use of fungicides to control late blight disease. Secondly, the study results suggest that the release of 3R-gene Shangi would generate the greatest economic benefits of KES 845.9 million (US$ 8.2 million) annually. The expected net benefits of 3R-gene Asante are KES 7.3 million (US$ 0.07 million) annually. 3R-gene Tigoni, with the lowest potential adoption rates, would be expected to realize a negative net of KES of -1.26 million (US$ -0.01 million) annually. Significant potential economic gains, which is expected to increase with better awareness of biotech crops, support the immediate release of 3R-gene Shangi in Kenya.

## Introduction

Agriculture continues to be the backbone of Kenya’s economy. The agricultural sector directly contributes to about 22% of Kenya’s GDP and indirectly provides an additional share through links with other sectors [1, 2]. The sector employs more than 70% of the rural workforce of Kenya and accounts for approximately 12% waged employment [2, 3]. Thousands of households in Kenya depend on agriculture for income and food security, with the majority of production being driven by smallholder farmers. The country’s agricultural growth, and in turn economic growth, depends on enabling smallholder farmers to achieve their potential in agricultural production. Additionally, enabling smallholder farmers to contribute more meaningfully to agricultural production will increase farmers’ income; help them achieve food security; and increase employment, particularly among women and youth.

In Kenya, potato (*Solanum tuberosum* L.) is a key food crop, with potato production and processing employing millions and contributing billions to the economy [4]. Potato offers great potential to increase incomes of smallholder farmers, who represent approximately 98% [5] of the 1.17 million potato farmers in the country [6]. Estimates from the 2021 social accounting matrix for Kenya [7] indicate that potato contributes approximately 8% of the value added in the agriculture sector and KES 50 billion annually to the economy, supporting about 3.5 million actors along the potato value chain [4]. Observations from the country’s food balance sheet place potato as Kenya’s second most important food staple, second after maize in gross production, and fourth in quantity consumed [2].

Despite its significant role in the diets of Kenyans, the performance of the potato per unit area has been declining over time. Data from the Statistical Database of the Food and Agricultural Organization of the United Nations show a decrease of about 51% in yield from 20 ton/ha in 2005 to 9.8 ton/ha in 2021, much lower than the potential attainable yield of up to 30-40 tons/ha [8–10]. The declining yield is mainly attributed to limited quality seeds, adverse weather conditions, and pests and diseases, particularly late-blight (LB) disease caused by *Phytophthora infestans*, and potato nematodes [4, 5, 11–13]. Potato growing areas are mainly characterized by high rainfall and cool temperatures making the environment conducive for LB disease [14].

LB is mainly controlled through fungicide applications [13], and not only threatens yield but also increases the cost of production and, in turn, affects the income-earning potential of the crop [5, 15]. Early evidence estimates global LB losses of approximately 16% annually [16]. In Kenya, estimated losses attributed to LB range from 30–70% annually, and in some cases 100% depending on the susceptibility of variety and weather conditions [14, 15, 17], but estimates are imprecise because of spotty damage data collection and issues with yield and yield losses measurements. Furthermore, not all Kenya production area was considered.

The effects of LB are aggravated by the excessive cost of fungicides, which limits their use; inefficient spraying techniques in terms of the number of treatments, amount of water used, and time of day during treatment; and late management practices, particularly among smallholder farmers with limited resources [5]. Furthermore, the use of latently infected seed tubers leads to early epidemics of LB disease that require early treatment and expensive short regimes of fungicide application [13]. The effects of LB disease are further exacerbated by new strains of *Phytophthora infestans* that are adaptive to resistant cultivars, further increasing use of fungicides [13]. Moreover, the indiscriminate use of fungicides not only results in economic losses but also favors the emergence of fungicide-resistant strains of the pathogen, while posing food safety, public health, and environmental concerns [4, 18–20].

Even with the challenges impacting crop production and a yearly yield of 9.8 ton/ha in 2021, which is less than a tenth of the total maize area harvested in Kenya, potato produces the most food per hectare. This makes the crop an asset in the fight against food insecurity as an important alternative source of starch that requires less space. In addition, its minimal cultivation area makes it ideal for solving income problems, especially among resource-poor agricultural households and women and youth with limited land access.

Biotech crops, also known as genetically engineered crops, have been shown to help farmers facing production challenges that affect crop yields such as weather stresses, pests, and diseases, and eventually impact positively food security, social, health, environmental, and economic outcomes [21, 22]. Recognizing the destructive nature of potato LB and the difficulties of stacking multiple resistance genes into an elite variety by conventional breeding, the International Potato Centre (CIP) and the Kenya Agriculture and Livestock Research Organization (KALRO), in collaboration with other partners, have developed and field-tested biotech potato varieties resistant to LB. This has been achieved through genetic engineering, where three LB resistance genes (3R-gene) from wild potato relatives have been stacked in potato varieties that are preferred by local farmers and consumers [23–25]. Recognizing that the late blight resistance trait can be introduced into potato varieties using classical plant breeding and genetic engineering techniques, in this paper, we refer specifically to the LB resistant potato stacked with 3R-gene developed using genetic engineering methods as the “3R-gene” potato varieties.

Durability of LB resistance is an important aspect of the 3R-s gene technology because *P*. *infestans* is well-known to overcome rapidly LB resistant varieties. The stacked biotech varieties are expected to have long term resistance to LB due to a purposive multi-pronged strategy. First, the *R* genes used in this technology were chosen to give broad spectrum resistance. This means that strains of *P*. *infestans* able to overcome are rare. Second, the 3 *R* genes have been introduced together. This approach imposes on *P infestans* the need to be able to overcome all 3 *R* genes at the same time. Last, should a strain of *P*. *infestans* emerge and cause significant economic damages, the defeated 3R-gene variety will be replaced immediately by another 3R-gene variety which is using different *R* genes that the resistant strain of *P*. *infestans* cannot overcome. Following this technology strategy, the durability of the resistance to LB is likely to be stable over a long period of time.

Before the release and dissemination of the 3R-gene potato, an economic assessment of adoption must be carried out to assess its potential. Although several ex-ante economic impact studies have been carried out to assess biotech food crops in Africa [21, 22, 26, 27], country and crop-specific assessments are important to provide locally based and context-specific answers. Furthermore, ex-ante studies on the impacts of biotech crops to help support evidence-based decision making are scarce in Kenya. For 3R-gene varieties, although there is evidence of the complete resistance to LB in field trials in various locations in Kenya, there is no documented empirical study of their potential economic benefit in the country.

To fill this gap, this study provides an ex-ante economic evaluation of the use of 3R-gene potato in Kenya before its release and dissemination. In the analysis, we consider the main LB management practices in potato-producing regions, a necessary step in providing better counterfactuals to existing potato farmers compared to on-farm trials to assess potential economic impacts. Potato experts contributed to the development of realistic economic estimates and assessments of the acceptability of 3R-gene potato. The implications of this study will be useful for the legal and regulatory review of 3R-gene potato in Kenya.

## Materials and methods

### Methodology and analytical approach

The selection of potato varieties for transformation to 3R-gene varieties was based on a combination of farmer- and consumer-preferred traits to allow immediate adoption and impact [23]. The selection was made in consultation with potato experts from agricultural research organizations, which led to the selection of three potato varieties as shown in Table 1. The variety Asante was also released in Uganda under the name Victoria where it became very popular until it became increasingly susceptible to LB disease.

**Table 1:** Selected potato varieties and their traits.

Variety	Year of release	Attributes
Asante	1998	Attainable yield of up to 35–45 tons/ha, good chipping, boiling, and mashing quality, reasonably tolerant of late blight.
Shangi	2015	Attainable yield of up to 30–40 tons/ha, early maturity, short dormancy, highly prolific, fast cooking, versatile use, i.e., it can be used for domestic consumption and processing into chips and crisps.
Tigoni	1998	Attainable yield of up to 35–45 tons/ha, good chipping, boiling and mashing quality, tolerant to late blight.

Source: [28, 29]

**Fig 1:**
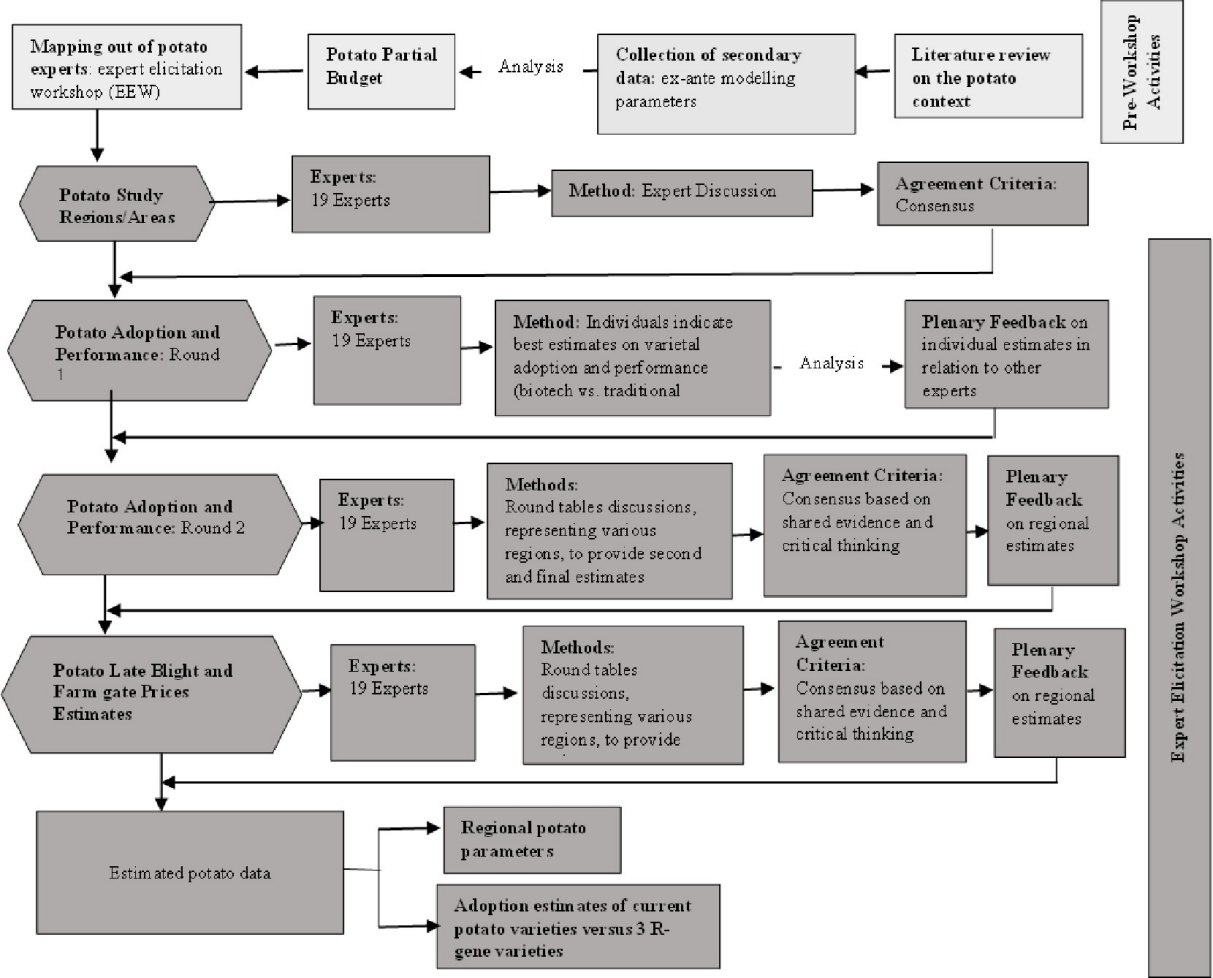
Potato expert elicitation workshop procedure.

The assessment of the potential economic benefits of the 3R-gene variety potato began with a review of relevant literature to understand the state of the potato subsector in the country. Secondary data was also collated on key parameters needed for the ex-ante analysis guided by the literature review. Based on the literature review and secondary data, a partial potato budget was developed to estimate the cost of potato production in the country. In-depth stakeholder engagement was conducted with various local potato experts and potato areas were classified into study regions. Additionally, relevant adoption estimates for potato varieties were obtained from stakeholders. Fig 1 provides an overview of the steps followed in involving the expert in the elicitation. Experts in the potato value chain included representatives of potato farmers, seed potato producers/multipliers, potato aggregators/distributors, extension officers, potato research institutions, local universities, local nonprofit organizations, and potato experts of the county and national governments.

Additional steps in the study included an ex-ante economic assessment training workshop for selected local potato experts, data analysis, and dissemination of findings.

To conduct an ex-ante economic analysis, various models were employed to evaluate the impact of agricultural technologies. These include the economic surplus model (ESM), cost–benefit analysis [30], linear programming model [31] and computable general equilibrium (CGE) models [32]. The choice of model depends on various factors including acceptable assumptions and available data.

The ESM is employed and widely used in various ex-ante studies [21, 22, 26, 27, 30, 33]. In this study, a multi-region ESM is implemented using the IFPRI DREAMpy tool [34] to assess the potential economic benefits of adopting 3R-gene potato technology in Kenya.

In the model, the adoption of agricultural technologies results in a reduction in the cost of production and/or changes in yields. Net effects lead to changes in the surplus of the consumer and the producer. The model is parsimonious and versatile in data and enables users to assess changes with and without 3R-gene potato technology in multiple geographical locations and considers spillovers [26, 27]. Since external trade is minimal, a closed economy is assumed in the analysis. A detailed underpinning of the multi-region ESM model is presented in [34] and thus not replicated here.

In addition to computing the economic surplus, this paper extends the results to assess the potential poverty reduction impacts following [35, 36].

The number of people lifted from poverty is given by

$$\Delta \;N_{p}=\left(\frac{\Delta ES}{Agriculture\;value\;added}\;x\;100\%\right)\;x\;\frac{\partial \;\ln\left(\frac{N_{p}}{N}\right)}{\partial \;\ln\left(Y\right)}\;x\;N_{p}$$

where Δ *N*_*p*_ is the number of poor lifted above the poverty line, *N*_*p*_ is the total number of poor, *N* is the total population, *Y* is agricultural productivity, and Δ*ES* is the change in economic surplus.

The measures proposed by [35, 36] have been widely applied in analyzing poverty effects in the adoption of new agricultural technologies [27, 37]. Furthermore, given that decision makers would be concerned about the uncertain irreversible costs with the release of biotech crops, a real options model is used to assess the ex-ante benefits and costs with the release of the 3R-gene potato in Kenya. Using the real option approach, the option of postponing or adopting an innovative technology considers the Maximum Incremental Social Tolerable Irreversible Costs (MISTICs) [38]. MISTICs acts as a threshold value that individuals are willing to incur as compensation for the benefits obtained from the technology [39]. The real options model has been used in similar studies to assess the benefits and costs of introducing biotech crops [27, 40].

### Data and parameter estimations

#### Data process

**Fig 2:**
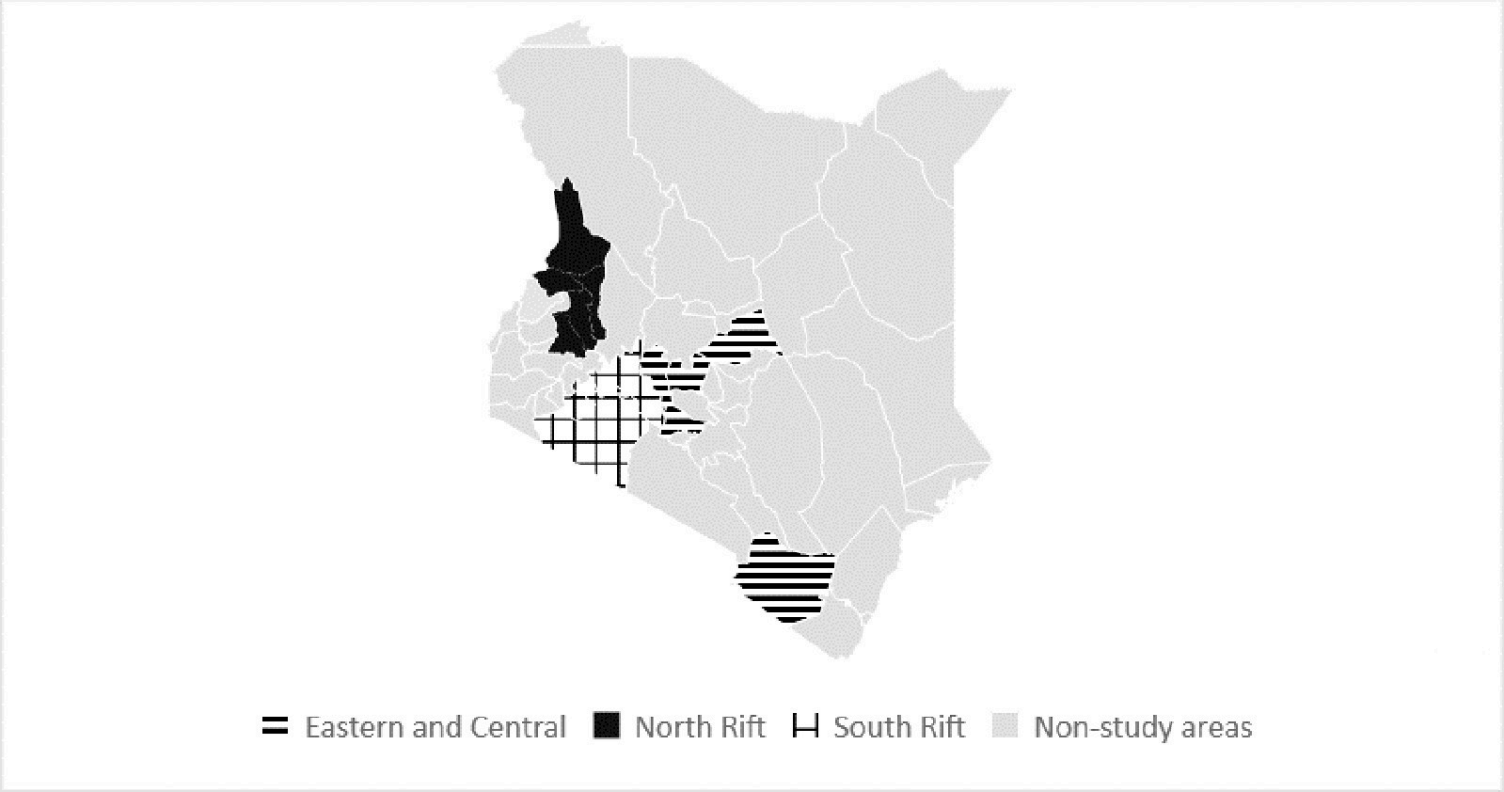
Potato study regions.

The analysis focused on the main areas of potato production in the country, classified by the NPCK as the North Rift Region, the South Rift Region, and the Eastern and Central Region (Fig 2).

**Fig 3:**
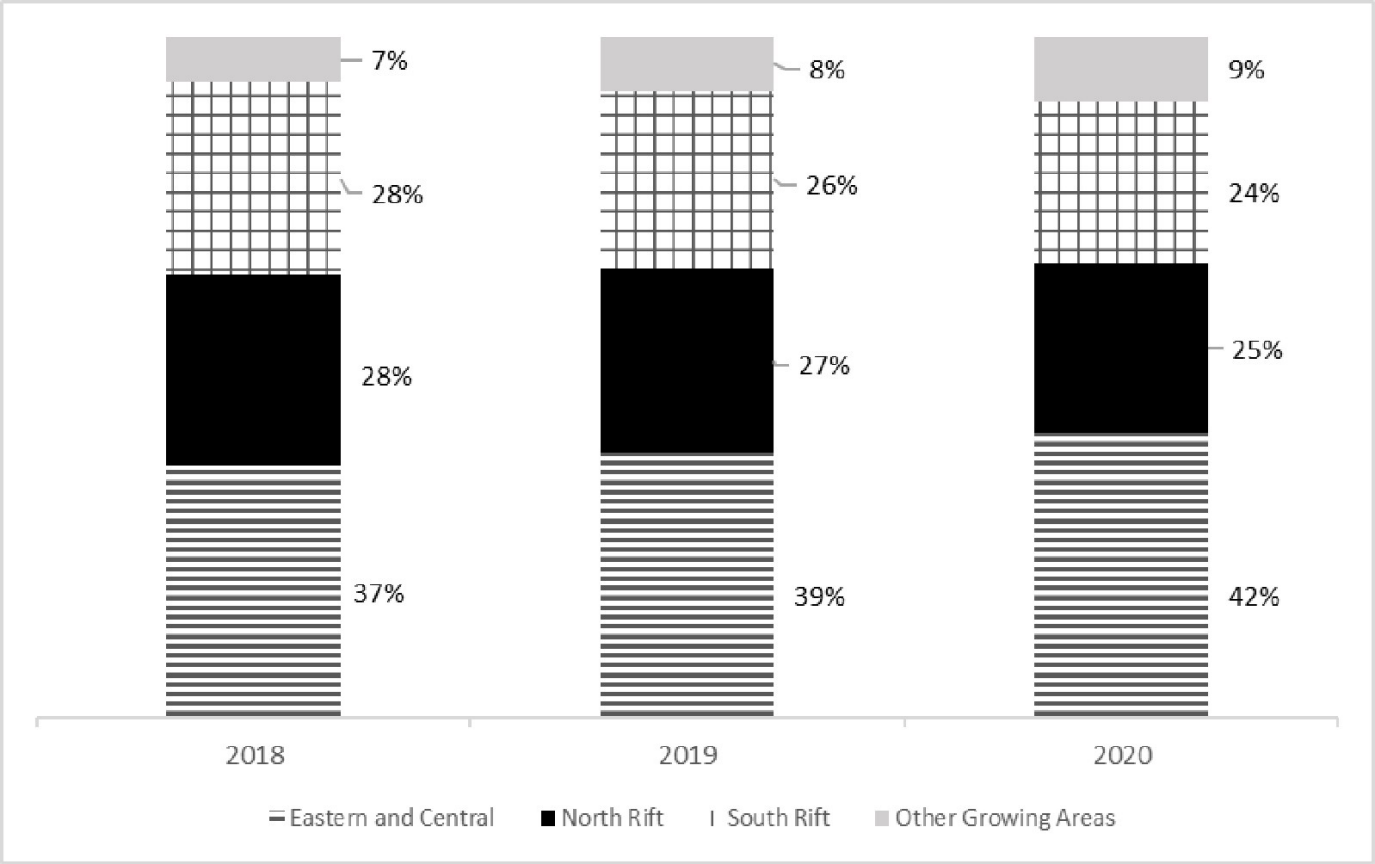
Share of potato production per region. Data Source (1).

Together, the areas make up over 90% of the potato production in the country (Fig 3).

Data used in the analysis were obtained from three main sources. Data on potato production performance and food balances were obtained from online statistical databases (KilimoSTAT, an Open Data Platform for the Ministry of Agriculture and Livestock Development, and FAOSTAT). Key parameters, such as real interest rates, demand and supply elasticities, and input prices and quantities, were obtained from published secondary sources. Potato varietal data and adoption estimates were obtained from potato experts during an elicitation workshop. Experts were identified along the potato value chain. The expert elicitation exercise was designed to not only elicit individual responses but also to facilitate interaction and discussions between experts. 

#### Parameter estimations

This section presents parameter estimates derived from secondary data and potato experts during the expert elicitation workshop.

**Fig 4:**
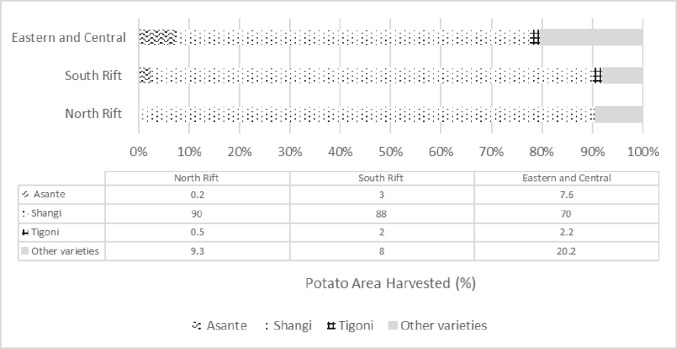
Current adoption rates. Source: Expert estimations.

*a) Adoption*. Expert estimates of adoption rates of existing potato varieties, measured as a percentage of the potato area harvested, indicate that Shangi is the most popular potato variety in all major production areas (Fig 4).

Table 2 presents the adoption rate estimates of the 3R-gene varieties of interest. Of the three adoption estimates elicited from experts, the ‘most likely’ adoption estimates are presented here.

**Table 2:** Expected adoption estimates.

Variety	Adoption Parameters	North Rift	South Rift	Eastern and Central
**Asante**	% area at maximum adoption	2	5	14
	Expected years to reach maximum adoption	5	7	5
	Expected years at maximum adoption	10	8	10
	Expected years to abandonment	3	6	8
**Shangi**	% area at maximum adoption	12	44	20
	Expected years to reach maximum adoption	5	7	5
	Expected years at maximum adoption	15	13	10
	Expected years to abandonment	5	9	5
**Tigoni**	% area at maximum adoption	1	4	5
	Expected years to reach maximum adoption	4	6	5
	Expected years at maximum adoption	4	4	7
	Expected years to abandonment	2	3	4

*Detailed estimates are presented in Table A in S1 Table.

Source: Expert Estimations

Adoption estimates differ between regions reflecting differences in production areas that are characterized by a wide range of conditions such as varietal preferences, availability of new varieties thorough the formal seed system, perception of local farmers of the impact on their market of 3R-gene potato, presence of established commercial farmers, and viable extension channels. These parameters appeared to explain perceived differences in likely adoption. The expected adoption area for biotech potato is largest for 3R-gene Shangi, ranging from 12% to 44% of the potato area in the country. The adoption rate of the 3R-gene Asante is estimated to range from 2 to 14%, while that of the 3R-gene Tigoni from 1% to 5%.

**Fig 5:**
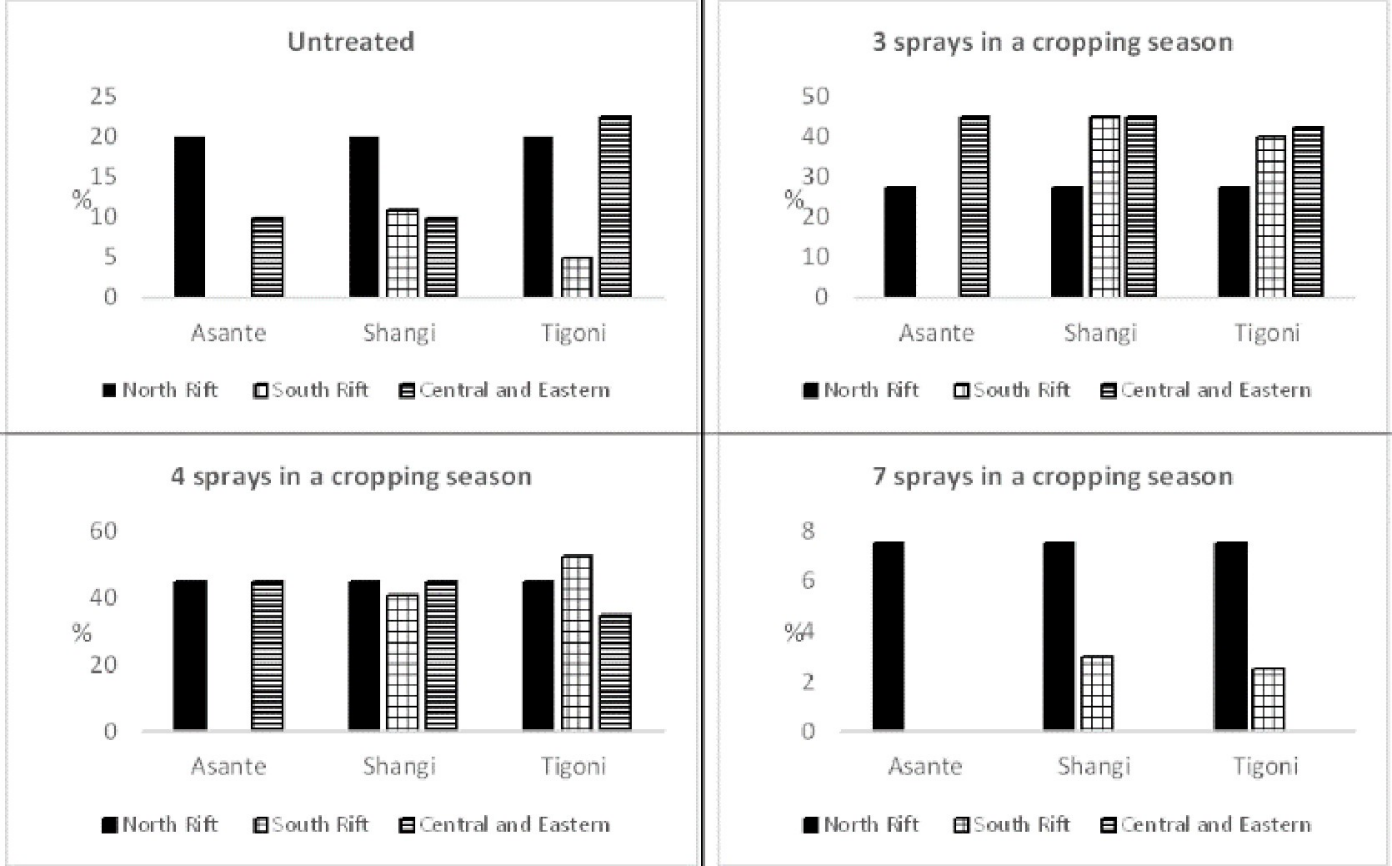
Late blight management practices (% of farmers). Source: Expert estimations.

*b) Late blight management*. The four main LB management regimes [41] in potato production regions were assessed. The regimes are: untreated (no fungicide application); triweekly fungicide application intervals (resulting in three sprays per cropping season); biweekly intervals (four sprays per cropping season); and weekly intervals (seven sprays per cropping season). Experts observed that most farmers apply three to four sprays per cropping season (Fig 5).

Potato yield varies with the different LB management practices, with high yields observed with weekly fungicide sprays during a growing season. Experts provided the expected yield changes with the adoption of 3R-gene potato relative to the non-3R-gene potato for the three varieties in the three potato production regions for each of the four LB management regimes (Table 3). On average, the production losses due to LB disease amount to 23% based on the data provided by experts, which were extrapolated to the total national production.

**Table 3:** Expected yield performance (annual averages; ton/ha).

		North Rift		South Rift		Eastern and Central	
	Variety	Current Yields	Expected Yields with 3R-gene Variety	Current Yields	Expected Yields with 3R-gene Variety	Current Yields	Expected Yields with 3R-gene Variety
**Untreated**	**Asante**	7	11			7	10
	**Shangi**	5	10	6	9	7	11
	**Tigoni**	7	11	7	7	5	8
**Triweekly Fungicide appl.**	**Asante**	10	11			11	12
	**Shangi**	9	11	16	19	12	14
	**Tigoni**	10	11	19	27	10	12
**Biweekly Fungicide appl.**	**Asante**	12	14			12	13
	**Shangi**	11	12	21	28	14	15
	**Tigoni**	12	14	26	37	11	12
**Weekly Fungicide**	**Asante**	16	16				
	**Shangi**	17	17	33	33		
	**Tigoni**	17	17	36	36		

Source: Expert estimations

*c) Production budget*. In terms of production costs, the costs related to pest and disease control would be reduced and are estimated to be between KES 96,661(US$ 937) per ha and KES 127,306 (US$ 1,234) per ha, with differences attributable mainly to the level of mechanization (Table 4).

**Table 4:** Production cost less fungicide costs.

Budget Components	Units	Value- County in South Rift Region	Value- County in Eastern and Central Region
Land preparation	KES/acre	4,800	3,600
Planting	KES/acre	22,000	35,800
Weeding	KES/acre	5,500	4,800
Foliar and application	KES/acre	400	640
Control of pests and diseases	KES/acre	9,100	4,680
Harvesting and Handling	KES/acre	4803	4800
Working capital	KES/acre	1,631	1,901
**Total cost per acre**	**KES/acre**	**39,134**	**51,541**
**Total costs per Ha**	**KES/Ha**	**96,661**	**127,306**

*Costs related to pests and diseases are assumed to include fungicide costs.

Source: [42]

The costs of fungicide application, including fungicide costs and labor, vary between areas, depending on the application rates and the number of fungicide formulations applied (Table 5).

**Table 5:** LB fungicide costs associated with the fungicide spray regime (KES per ha).

LB Fungicide Spraying Regime	Season	North Rift	South Rift	Eastern and Central
Untreated	No LB fungicide sprayed in a cropping season			
Triweekly Fungicide Appl.	Long rains	15,500	19,500	9,386
	Short rains	15,500	9,750	9,386
Biweekly Fungicide Appl.	Long rains	19,500	27,500	12,844
	Short rains	19,500	13,750	12,844
Weekly Fungicide Appl.	Long rains	31,500	56,000	
	Short rains	31,500	28,000	

Source: Expert estimations

Adoption of 3R-gene varieties would eliminate the costs of fungicide applications, leading to cost changes ranging from -7% to -30% per ha among farmers applying fungicides (Table 6).

**Table 6:** Cost changes (%) with 3R-gene variety adoption (per ha).

	North Rift	South Rift	Eastern and Central
No. fungicide application	0	0	0
Triweekly fungicide appl.	-14	-13	-7
Biweekly spraying costs	-17	-18	-9
Weekly fungicide appl.	-25	-30	

Source: Authors’ own estimation using expert opinions

*Negative values imply a cost reduction with the adoption of 3R-gene variety

On average, the adoption of 3R-gene varieties lead to a cost saving of 12% relative to the production of a non-3R-gene variety.

**Fig 6:**
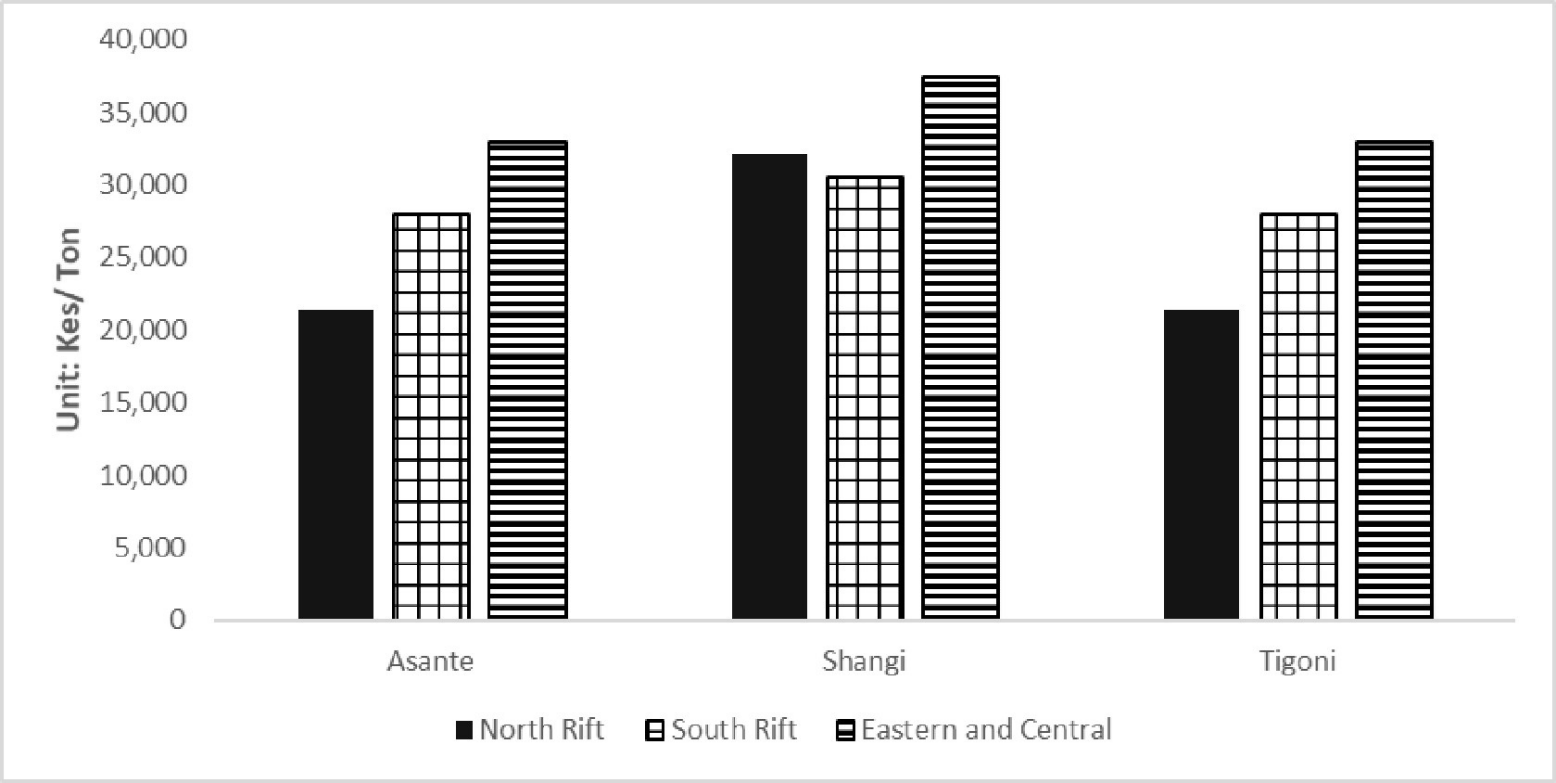
Potato farm gate prices. *Values displayed are for estimates of the ’most likely’ scenario. Detailed estimates are provided in Table B in S1 Table. Source: Expert estimations.

Fig 6 shows the estimated farm gate prices by region and variety. Farm gate prices range from KES 21, 429/ton (US$ 208/ton) to KES 33,000/ton (US$ 320/ton).

**Fig 7:**
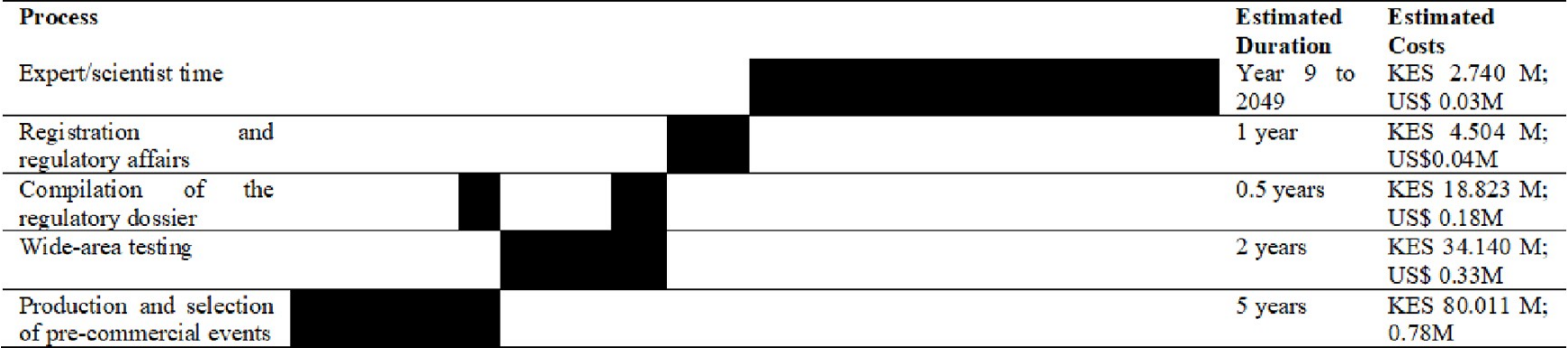
Research and development costs. * Process 3 overlaps partly with Processes 1 and 2. * Maintenance research costs are estimated to be 2% of total costs. Source: Compiled by authors based on (2).

*d) Research and development costs*. Research and development costs for the development of biotech potato are determined from CIP estimates and modified to include maintenance and research costs [43] (Fig 7). It is worth mentioning that these are estimates and may be subject to change depending on regulatory requirements.

*e) Additional baseline parameters*. Additional parameters are presented in Table 7.

**Table 7:** Additional key baseline parameters.

	Parameter	Unit	Value	Source
General	Base year	date	2020	Expert elicitation
	Simulation period	years	30	Based on adoption estimations
	Discount rate	%	11.5	Economic Opportunity Cost of Capital (EOCK)- [44]
	Official exchange rate	KES per 1US$	103.2	Source: World Bank, [45] average period 2018-2020
Market	Growth in production supply	%	4.4	FAOSTAT [9]– 2018-2020 average
	Growth in consumption demand	%	5.16	FAOSTAT[9] – Growth of food supply quantity (kg / capita / year) 2018-2020 average
	Elasticity supply		0.596	[46]
	Demand elasticity		−0.893	[47]
Technology	R&D time lag (years)	years	5-8	Based on [43]
	R&D success probability (%)		90	Expert opinion

## Results

### DREAMpy model

**Fig 8:**
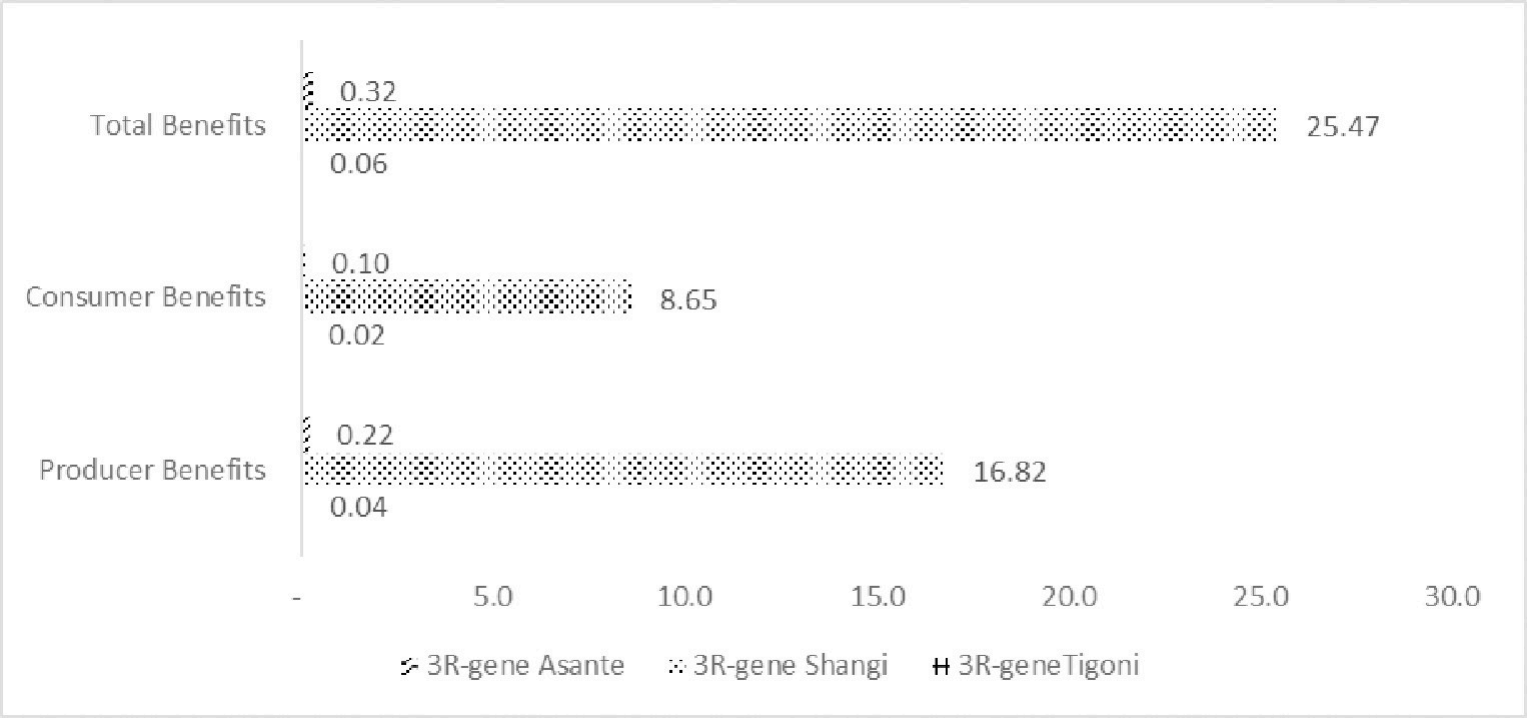
Present value of R&D total benefits (billion KES).

**Fig 9:**
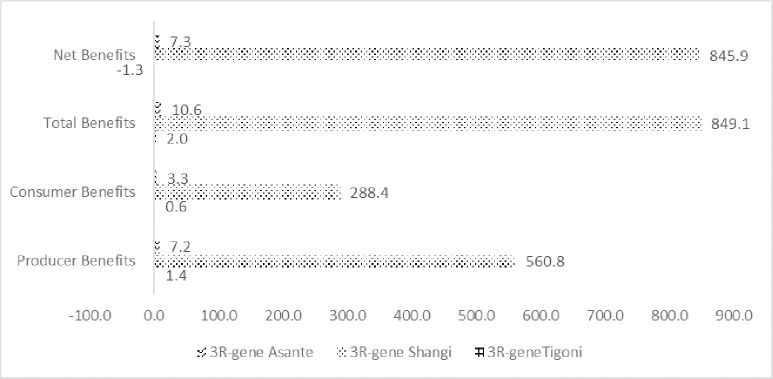
Present value of R&D benefits (million KES, annual).

Estimates for the economic surplus for the three varieties of interest in the main potato production regions in Kenya for a 30-year period are estimated using the DREAMpy model. The results are presented for various scenarios (Table 8; Figs 8 and 9).

**Table 8:** Economic surplus estimates of 3R-gene Shangi. Average annual present values in KES million.

	Region	Δ Producer Surplus	Δ Consumer Surplus	Δ Total Surplus	ΔCosts	ΔB−ΔC	ΔB/ΔC	IRR
No spraying	North Rift	11.1	8.8	19.8	0.1	19.7	168.0	78
	South Rift	25.2	6.9	32.1	0.1	32.0	217.2	76
	Eastern and Central	15.2	9.8	25.0	0.1	24.9	188.2	83
	Total	51.5	25.4	76.9	0.4	76.5	193.0	79
Triweekly Fungicide Application	North Rift	5.6	10.2	15.8	0.2	15.6	97.1	69
	South Rift	148.8	39.3	188.1	0.6	187.5	311.0	83
	Eastern and Central	23.6	41.8	65.5	0.6	64.9	109.5	74
	Total	178.0	91.4	269.4	1.4	268.0	197.4	78
Biweekly Fungicide Application	North Rift	(11.2)	29.7	18.5	0.3	18.2	69.5	64
	South Rift	328.9	64.6	393.5	0.6	392.9	714.0	98
	Eastern and Central	(8.2)	66.4	58.2	0.6	57.6	97.4	73
	Total	309.5	160.7	470.1	1.4	468.7	332.4	86
Weekly Fungicide Application	North Rift	(0.0)	5.6	5.5	0.0	5.5	124.8	74
	South Rift	21.8	5.3	27.2	0.0	27.1	673.7	97
	Eastern and Central							
	Total	21.8	10.9	32.7	0.1	32.6	386.4	88
	Overall total	560.8	288.4	849.1	3.3	845.9	260.3	

The first scenario considers a single release of a 3R-gene potato variety where performance is compared among the three varieties of interest: the 3R-gene Asante, Shangi, and Tigoni. It should be noted that the benefits from the three varieties cannot be aggregated because the assumption underlying the assessment is that the release of 3R-gene potato varieties is pursued one at a time rather than all varieties at the same time.

Generally, the results indicate that the adoption of 3R-gene potato accrues benefits to the country (Fig 8). The greatest share of benefits is captured by producers.

Although the annual present value of the benefits for the 3R-gene Asante and 3R-gene Tigoni varieties are positive, the net benefits for 3R-gene Tigoni are negative, KES -1.3 million (US$ -0.01 million), and that of -gene Asante is just over KES 7 million (US$ 0.07 million) (See Tables A and B in S2 Table). The greatest economic surplus is generated under the 3R-gene Shangi, which has an annual discounted net value of KES 845.9 million (US$ 8.2 million).

The performance of 3R-gene Asante and Tigoni relative to 3R-gene Shangi is the result of the low adoption values (see Table 3) that lead to gains that are not large enough to compensate for the cost of technology.

The distribution of 3R-gene Shangi benefits across the regions shows that the largest benefits would accrue in the South Rift Region, which has the largest adoption area (Table 8). In addition, the share of benefits among farmers due to their fungicide spraying practice is largest under the biweekly application of fungicides, which constitutes the largest share of potato producers according to estimates from experts.

**Fig 10:**
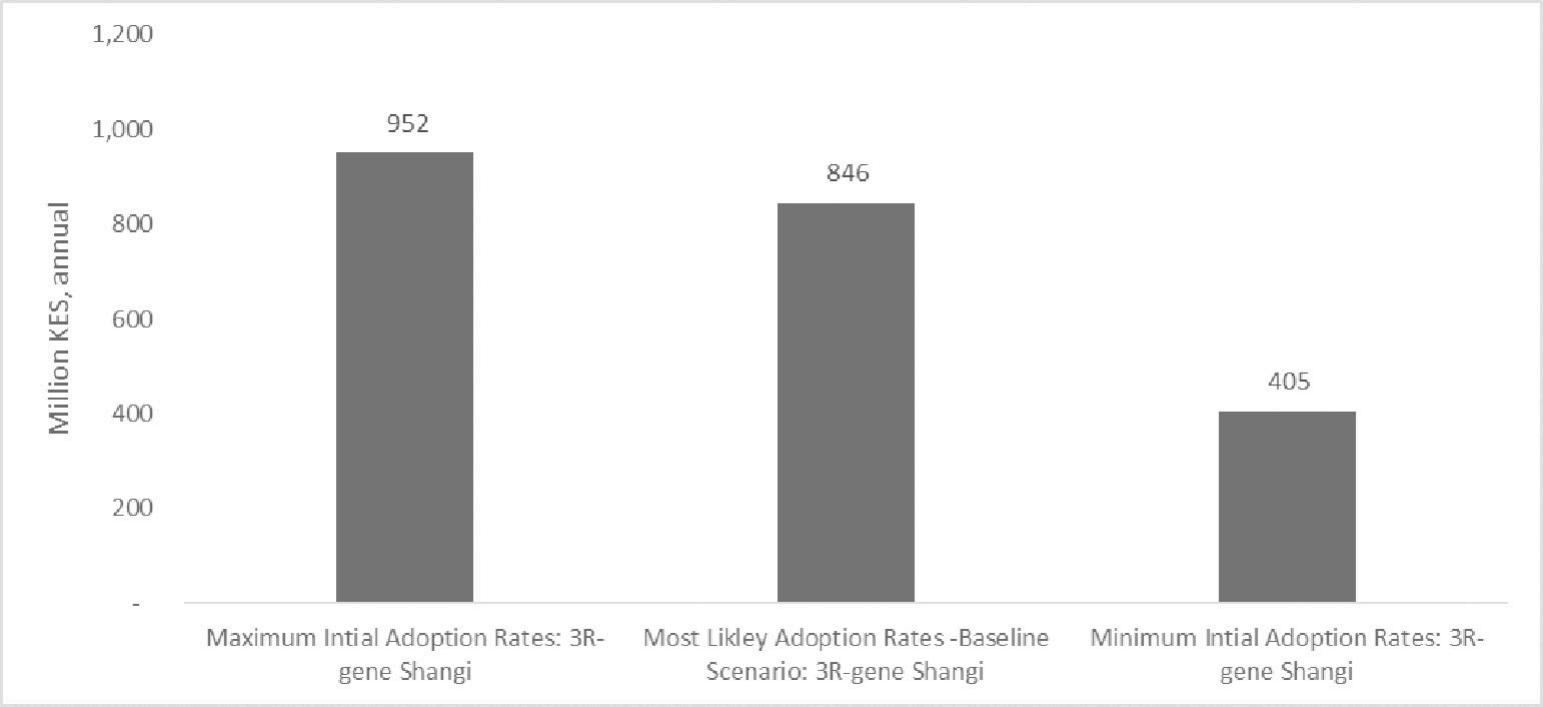
Adoption rate scenario. Net annual benefits relative to baseline value.

In addition to the 3R-gene Shangi baseline scenario based on the most likely adoption rate, net benefits based on maximum and minimum adoption rates are presented in Fig 10. By performing the scenario, we can assess the sensitivity of the results to adoption, a key input parameter, and provide estimates of the potential variation in expected benefits.

The results from the maximum and minimum adoption rate scenarios are from deterministic runs of the model where values were obtained from experts (see Table A in S1 Table). The results reveal that the net annual economic benefits are 13% above the baseline level with maximum adoption rates and 52% lower with the minimum adoption rates.

**Fig 11:**
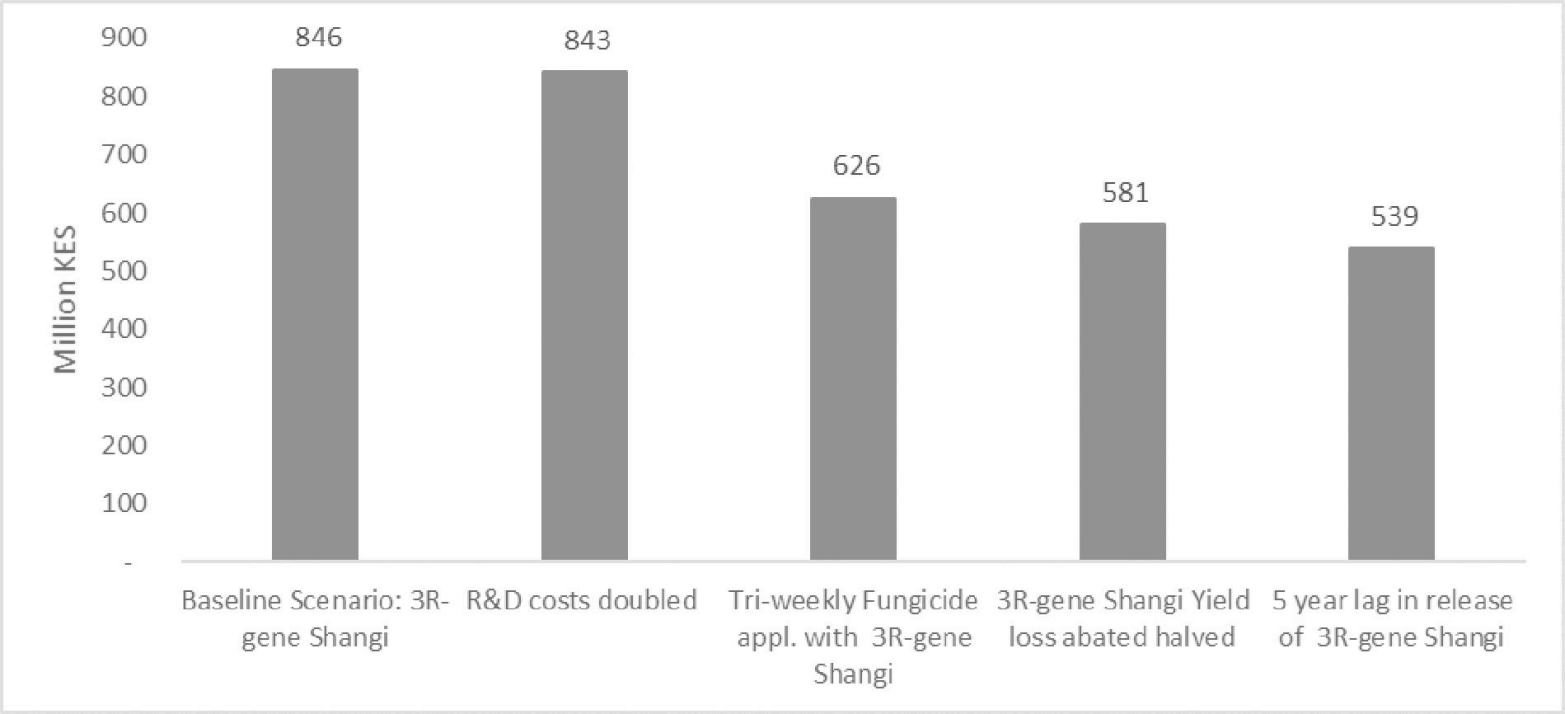
Scenario summaries. Net annual benefits relative to baseline value.

Four additional scenarios departing from the baseline scenario are worth presenting to assess the plausible lowest value of net economic benefits with 3R-gene Shangi: (1) doubling of the R&D costs such as excessive regulatory and monitoring requirements; (2) continuation of tri-weekly fungicide application by some farmers for some time; (3) yield loss abated only by half due to the release of a lower yielding 3R-gene Shangi; and (4) a five-year lag in the release of 3R-gene Shangi (Fig 11).

The scenario of doubled research and development costs, which could arise from government regulation and maintenance research costs, results in lower net benefits by 0.4% compared to the baseline level.

While the adoption of 3R-gene Shangi eliminates the need for LB fungicide application, it is reasonable to expect some farmers who commonly use fungicides to continue with LB fungicide application at least for some time. The results show that the economic benefits of fungicide application (three sprays per cropping season) are 26% lower than the baseline level. Proper information on the management practices of the 3R-gene Shangi will reduce the likelihood of this scenario.

An assumption that farmers achieve only half of the yield loss estimated by experts with the adoption of 3R-gene Shangi leads to economic benefits lower than the baseline level by 31%. This highlights how important it is to release a 3R-gene Shangi that is identically yielding to the conventional Shangi variety.

The lowest benefits are likely to arise with a five-year lag in the release of 3R-gene Shangi, which could occur due to delays in concluding the R&D and regulatory processes. Such an occurrence is likely to reduce expected net annual benefits by 36% to KES 539 million (US$ 5.2 million).

A crucial finding during the expert elicitation exercise on the adoption of 3R-gene potato varieties is the growing interest in potato varieties currently gaining popularity in the country (Wanjiku, Unica, and Sherekea). As a result, an additional scenario is estimated to assess the expected benefits should a new 3R-gene potato variety be released within the simulation period. The assumption made is that after 10 years of its release and commercialization, the 3R gene Shangi will be displaced by the ‘new’ 3R-gene variety. Thus, the two releases are mutually exclusive though pursued within the period of analysis.

Given its superior qualities relative to 3R-gene Shangi, it is assumed that the new 3R-gene variety adoption values would be higher than those of 3R-gene Shangi. Therefore, the optimistic expert adoption estimates of 3R-gene Shangi are used to estimate the benefits of the new 3R-gene variety. The adoption of two 3R-gene varieties is assumed to be consecutive to each other, with the new 3R-gene variety being released after 3R-gene Shangi has been abandoned.

**Fig 12:**
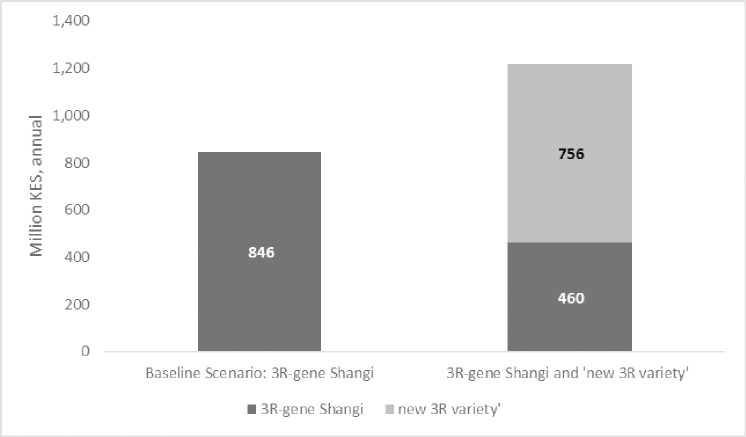
Summary of annual benefits with two consecutive 3R-gene potato releases relative to baseline values.

The total net benefit increases with two consecutive releases of popular varieties compared to a single release during the same period (Fig 12). The results support gradual release of 3R-gene potato varieties, consistent with farmers’ preferences at the time.

With a total of 1.17 million potato farmers, we estimated the potential number of people who can be lifted out of poverty in each of the 3R-gene Shangi scenarios assessed in the previous section (Table 9).

**Table 9:** Estimated poverty effects of the different 3R-gene Shangi scenarios.

Scenario	Total net ES (billion KES)	Average benefit per year (billion KES)	Gains from R&E as % of agricultural production (annual basis)	Poverty elasticity	Poverty reduction as % of the poor (all years)	Poverty reduction as % of the poor (yearly basis)	Number of poor escaping poverty (all years)	Number of poor who escape poverty (annual basis)
3R-gene Shangi baseline	25.376	0.846	0.033	0.570	0.006	0.0002	91,884	3,063
Triweekly fungicide application with 3R-gene Shangi	18.784	0.626	0.024	0.570	0.004	0.0001	68,013	2,267
5-year lag in release	16.171	0.539	0.021	0.570	0.004	0.0001	58,554	1,952
R&D costs doubled	25.279	0.843	0.033	0.570	0.006	0.0002	91,530	3,051
Yield loss abated halved	17.432	0.581	0.023	0.570	0.004	0.0001	63,120	2,104
3R-gene Shangi and new 3R-gene variety	36.472	1.216	0.047	0.570	0.008	0.0003	132,059	4,402

The estimated effects on the number of people lifted from poverty range from 1,952 to 4,402 on an annual basis. The effects depend on the size of the total net economic surplus (ES). Our estimate over the cumulate life span of the 3R-gene Shangi, at the baseline, is that 91,884 of today’s poor population will escape poverty.

### Real options model

A real options model is also used to assess ex-ante benefits and costs with the release of 3R-gene potato in Kenya. To address the issues of irreversibility and uncertainty we estimated the Social Incremental Reversible Benefits (SIRBs) and Maximum Incremental Social Tolerable Irreversible Costs (MISTICs) for the 3R-gene Shangi in Kenya. These presented are in Tables 10 and 11, respectively.

**Table 10:** SIRBs for 3R-gene potato in Kenya at 11.5% discount rates in Kenyan Shillings.

		SIRB					
Type of farmer	Potato region	Total (billion)	per year (billion)	per ha (million)	per ha and year (million)	per household /year	per farmer/year
No spraying	North Rift	7.77	0.41	9.10	0.48	317.42	18,976.15
	South Rift	11.78	0.63	5.45	0.29	599.91	36,483.02
	Eastern and Central	12.26	0.65	9.80	0.52	373.59	27,058.97
Triweekly Fungicide Application	North Rift	6.71	0.36	5.71	0.30	274.02	7,358.60
	South Rift	55.91	2.97	6.32	0.34	2,846.64	15,869.09
	Eastern and Central	28.30	1.51	5.03	0.27	862.27	7,647.60
Biweekly Fungicide Application	North Rift	5.92	0.31	3.08	0.16	241.57	3,063.42
	South Rift	112.38	5.98	13.95	0.74	5,721.99	26,674.50
	Eastern and Central	18.45	0.98	3.28	0.17	562.32	4,313.34
Weekly Fungicide Application	North Rift	0.60	0.03	1.88	0.10	24.56	1,209.07
	South Rift	1.47	0.08	2.50	0.13	75.10	3,091.43
	Eastern and Central	-	-	-	-	-	-
	Country	254.46	13.54	6.29	0.33	1,114.73	11,568.56

**Table 11:** MISTICs for 3R-gene potato in Kenya at 11.5% discount rates in Kenyan Shillings.

		MISTIC					
Farmers	Potato region	Total (billion)	per year (billion)	per Ha (million)	per ha and year (million)	per household /year	per farmer/year
No spraying	North Rift	6.12	0.33	7.16	0.38	249.75	14,931.07
	South Rift	9.27	0.49	4.29	0.23	472.03	28,706.06
	Eastern and Central	9.65	0.51	7.71	0.41	293.96	21,290.90
Triweekly Fungicide Application	North Rift	5.28	0.28	4.49	0.24	215.61	5,789.99
	South Rift	43.99	2.34	4.98	0.26	2,239.83	12,486.33
	Eastern and Central	22.26	1.18	3.96	0.21	678.46	6,017.38
Biweekly Fungicide Application	North Rift	4.66	0.25	2.42	0.13	190.08	2,410.40
	South Rift	88.42	4.70	10.98	0.58	4,502.26	20,988.39
	Eastern and Central	14.52	0.77	2.58	0.14	442.45	3,393.88
Weekly Fungicide Application	North Rift	0.47	0.03	1.48	0.08	19.32	951.34
	South Rift	1.16	0.06	1.97	0.10	59.09	2,432.44
	Eastern and Central	-	-	-	-	-	-
Country		200.22	10.65	4.95	0.26	877.11	9,102.53

The SIRBs in the study regions are significant, ranging between KES 0.03 billion (US$ 0.31 million) and KES 5.98 billion (US$ 57.90 million) per year, and vary by region and type of farmer (Table 10). The SIRBs are an indication of the benefits that the country will forgo by delaying the release of 3R-gene Shangi. At the national level, the results can be interpreted as implying that delaying the release of 3R-gene Shangi for another year will deny the country KES 13.54 billion (US$ 131.11 million) yearly.

Since SIRBs are highly sensitive to the discount rate used to discount future benefits, we investigate the benefits variations for a ±2% of discount rates. A variation of the discount rate of +2% leads to the annual SIRBs decreasing by 20% and increasing by 29% with a variation of -2% at the national level. The results of the sensitivity analysis do not vary the main finding of the potential benefits of the 3R-gene Shangi in Kenya.

The hurdle rate measures the rate at which the social incremental reversible benefits plus the irreversible benefits must be higher than the social incremental irreversible costs to justify the immediate release of a new technology. The hurdle rate estimated using potato yield data, is 1.271. In other words, the results indicate that the social irreversible costs associated with 3R-gene Shangi should be lower than the benefits by 1.271, to justify the release of the technology.

MISTICs in regions and farmers range between KES 0.03 billion (US$ 0.24 million) and KES 4.70 billion (US$ 45.56 million) per year (Table 11). These indicate the threshold values below which the social incremental irreversible costs must be for 3R-gene Shangi to have positive welfare impacts.

The results of MISTICs per farmer per year provide a good case for the immediate release of 3R-gene Shangi in Kenya with farmers willing to tolerate incremental social irreversible costs as high as KES 28,706 (US$ 278.03) per year as compensation for the benefits obtained from the technology. The distribution of MISTICs across a much larger group, in this case households in various regions and the country, yields much lower values. The results indicate that farmers have a much higher interest in 3R-gene Shangi compared to households. At the country level, the results indicate that 3R-gene Shangi should not be released if households are willing to pay more than KES 877.11 (US$ 8.50) per year not to have 3R-gene Shangi released in the country.

A sensitivity analysis of ±2 of the discount rates leads the annual MISTICs decreasing by 15% with a variation of +2% and increasing by 14% with a variation of -2% at the national level. The analysis is extended to a sensitivity analysis for the risk free rates. Higher risk free rates of 2% and 4%, associated with higher opportunity costs and in turn hurdle rates, decrease annual MISTICs by 7% and 17% respectively.

## Discussion and conclusion

The result of this study supports the findings of ex ante economic impact assessment studies on economic benefits of biotech crops in Africa [21, 22, 26]. The findings suggest high returns with the adoption of 3R-gene Shangi, which is estimated to have the highest potential adoption rate based on experts’ experience of the variety’s superior qualities relative to 3R-gene Asante and 3R-gene Tigoni. The willingness to adopt 3R-gene Shangi, and in turn the expected higher benefits are attributed to its resistance to late blight and the excellent traits of the non-3R-gene, which are retained in the transformed variety. Similar observations are made in [48], where the benefits of agricultural biotechnology are greater with larger adoption areas.

The changes in producer surplus are approximately twice as large as the consumer surplus. The distribution of benefits is similar to that in agricultural studies [22], in which the demand elasticity (in absolute terms) is larger than the supply elasticity. We note that while producers are expected to benefit less in a closed economy from an increased supply that tends to suppress the domestic price relative to a small open economy, the increase in potato demand in Kenyan case [4] is likely to suppress a downward price change.

To put the potential benefits of the 3R-gene potato in relatable terms, our calculations suggest that 3,063 people will be lifted out of poverty with the adoption of 3R-gene Shangi annually, with a total of 91,884 individuals escaping poverty during the expected lifetime of the 3R-gene Shangi cultivation period. This reflects the potential economic power of the 3R-gene potato.

It is important to note the limitations of ESM when interpreting the results. The economic surplus model is sensitive to changes in key assumptions, mainly estimated yield and cost changes, and elasticities [26, 27]. In addition, while yield and cost changes are based on average values at the regional level, the assumed changes do not consider heterogeneity among farmers in the region [30]. This paper has attempted to overcome these shortcomings through sensitivity analysis, using various scenarios, and assumptions informed by an extensive literature review, national statistics and parameters, and expert views.

In the scenarios evaluated, the investigated effects of a delay in approval shows the greatest negative impact on the potential benefits of adopting the 3R-gene potato. Research, development, and regulatory delays reduce the benefits by 36%, but do not alter the importance and positive impact of 3R-gene Shangi. It is therefore prudent that regulatory efficiency and effectiveness be enhanced to realize the maximum benefits of the technology to Kenya’s producers and consumers.

Apart from sensitivity to key parameters, the modeled changes in the supply within the ESM does not take into account the transactional costs that are likely to occur with the release of agricultural technologies [26, 27, 30]. Transactional costs can be included after the supply shift impacts have accrued, reducing the level of benefits. Transaction costs such as those associated with dissemination, promotion, and adoption of the 3R-gene potato have not been considered here, given that they are not part of the R&D process of the not-for-profit and local developing institutions involved, and we continue to be interested in the returns on R&D investment to all developers. However, to mitigate the possibility of benefit overestimation, conservative assumptions about R&D costs and cost changes were made, in line with similar studies [30].

A robustness check of the estimated ESM benefits was performed using the real options model. Although no plausible risk of cultivating and consuming 3R-gene potato varieties has been identified, the real options model allows us to evaluate the economic benefits of the 3R-gene potato under conditions of irreversibility and uncertainty regarding their future benefits and costs. The 1.271 hurdle rates calculated using the real options approach indicate that 1 KES of incremental irreversibility costs needs to be compensated by about KES 1.271 of social incremental reversible and irreversible benefits [40]. Furthermore, the results indicate that if Kenya delays the release of 3R-gene Shangi, the country would be losing additional benefits of about KES 13.54 billion (US$ 131.11 million) annually.

Our estimates of the potential economic benefits of 3R-gene might have underestimated the potential benefits by ignoring the indirect benefits of the technology. Nevertheless, with potato LB disease remaining a severe threat to potato performance in Kenya, the results show its adoption has great potential to improve consumer and producer surplus, greatly affecting food security and the profitability of smallholder farmers.

Kenyan experts, including those who specialize in potato production, anticipate that outreach, communication and other awareness programs on the potential benefits of the 3R-gene potato are likely to stimulate higher adoption rates and increase benefits to producers and consumers in Kenya, especially with a widely disseminated and damaging disease such as late blight. In general, these findings provide a significant step towards the release, dissemination, and adoption of 3R-gene potato in Kenya. The results substantially improve our understanding of the potential economic performance of 3R-gene potato in Kenya and will help ensure that the enabling environment in the country continues to be conducive to valuable and safe technologies for Kenyan farmers to improve their food security.

## Supporting information

S1 Table Parameter estimates. (DOCX)

S2 Table DREAMpy scenario estimates. (DOCX)
