# Supplementary material for: Ex ante economic impact assessment of the 3R-gene potato in Kenya
Source: PLoS One. 2025 Mar 31;20(3):e0309329. doi: 10.1371/journal.pone.0309329 (PMC11957266; doi:10.1371/journal.pone.0309329)
Supplement: S1 Table — (DOCX) [file pone.0309329.s001.docx]

**Ex ante economic impact assessment of the 3R-gene potato in Kenya**

Evelyne Kihiu^1^*, Marc Ghislain^1^, Anthony Mwangi Kibe^2^, Ng’ang’a Nancy^3^, Marcel Gatto^4^, Jose B. Falck-Zepeda^5^

^1^International Potato Center (CIP), Nairobi, Kenya

^2^Department of Crops, Horticulture and Soil, Egerton University, Njoro, Kenya

^3^Kenya Agricultural Livestock and Research Organization-Tigoni, Limuru, Kenya

^4^International Potato Center, Hanoi, Vietnam

^5^International Food Policy Research Institute (IFPRI), Washington, USA

*Corresponding author

E-mail: [Evelyne.kihiu@cgiar.org](mailto:Evelyne.kihiu@cgiar.org) (EK)

**S 1 Table. Parameter estimates**

**Table A.** Adoption estimations

**Table B.** Average farm gate prices (Unit: KES/ton)

**Table A.** Adoption estimations

|  |  | **Adoption Parameters** |  | **North Rift** | **South Rift** | **Eastern and Central** |
| --- | --- | --- | --- | --- | --- | --- |
| **Shangi** | **3R-gene** | % area at maximum adoption | Min | 8 | 30 | 10 |
|  |  |  | Most likely | 12 | 44 | 20 |
|  |  |  | Max. | 17 | 53 | 30 |
|  |  | Expected years to reach maximum adoption | Min | 4 | 5 | 3 |
|  |  |  | Most likely | 5 | 7 | 5 |
|  |  |  | Max. | 5 | 10 | 7 |
|  |  | Expected years at maximum adoption | Min | 3 | 8 | 5 |
|  |  |  | Most likely | 15 | 13 | 10 |
|  |  |  | Max. | 22 | 15 | 15 |
|  |  | Expected years to abandonment | Min | 2 | 6 | 3 |
|  |  |  | Most likely | 5 | 9 | 5 |
|  |  |  | Max. | 10 | 15 | 7 |
|  | **Existing Technologies** | **Percentage of Area Harvested** |  | 90 | 88 | 70 |
| **Tigoni** | **3R-gene** | % area at maximum adoption | Min | 0 | 1 | 2 |
|  |  |  | Most likely | 1 | 4 | 5 |
|  |  |  | Max. | 1 | 6 | 7 |
|  |  | Expected years to reach maximum adoption | Min | 1 | 3 | 3 |
|  |  |  | Most likely | 4 | 6 | 5 |
|  |  |  | Max. | 6 | 8 | 6 |
|  |  | Expected years at maximum adoption | Min | 1 | 2 | 4 |
|  |  |  | Most likely | 4 | 4 | 7 |
|  |  |  | Max. | 6 | 7 | 9 |
|  |  | Expected years to abandonment | Min | 1 | 2 | 3 |
|  |  |  | Most likely | 2 | 3 | 4 |
|  |  |  | Max. | 3 | 4 | 5 |
|  | **Existing Technologies** | **Percentage of Area Harvested** |  | 1 | 2 | 2 |
| **Asante** | **3R-gene** | % area at maximum adoption | Min | 0 | 4 | 6 |
|  |  |  | Most likely | 2 | 5 | 14 |
|  |  |  | Max. | 5 | 5 | 15 |
|  |  | Expected years to reach maximum adoption | Min | 2 | 3 | 3 |
|  |  |  | Most likely | 5 | 7 | 5 |
|  |  |  | Max. | 6 | 10 | 7 |
|  |  | Expected years at maximum adoption | Min | 2 | 5 | 7 |
|  |  |  | Most likely | 10 | 8 | 10 |
|  |  |  | Max. | 12 | 13 | 11 |
|  |  | Expected years to abandonment | Min | 2 | 3 | 5 |
|  |  |  | Most likely | 3 | 6 | 8 |
|  |  |  | Max. | 4 | 9 | 10 |
|  | **Existing Technologies** | **Percentage of Area Harvested** |  | 0 | 3 | 8 |

Source: Expert estimations

**Table B.** Average farm gate prices (Unit: KES/ton)

|  |  | Shangi | Tigoni | Asante |
| --- | --- | --- | --- | --- |
| North Rift | Min | 23,571 | 10,714 | 10,714 |
|  | Most likely | 32,143 | 21,429 | 21,429 |
|  | Max. | 39,286 | 39,286 | 39,286 |
| South Rift | Min | 14,000 | 11,500 | 11,500 |
|  | Most likely | 30,500 | 28,000 | 28,000 |
|  | Max. | 42,500 | 35,000 | 35,000 |
| Eastern and Central | Min | 22,000 | 19,000 | 19,000 |
|  | Most likely | 37,500 | 33,000 | 33,000 |
|  | Max. | 61,000 | 58,000 | 58,000 |

*Values displayed are for estimates of the 'most likely' scenario.

Source: Expert estimations
