# Supplementary material for: Ex ante economic impact assessment of the 3R-gene potato in Kenya
Source: PLoS One. 2025 Mar 31;20(3):e0309329. doi: 10.1371/journal.pone.0309329 (PMC11957266; doi:10.1371/journal.pone.0309329)
Supplement: S2 Table — (DOCX) [file pone.0309329.s002.docx]

**Ex ante economic impact assessment of the 3R-gene potato in Kenya**

Evelyne Kihiu^1^*, Marc Ghislain^1^, Anthony Mwangi Kibe^2^, Ng’ang’a Nancy^3^, Marcel Gatto^4^, Jose B. Falck-Zepeda^5^

^1^International Potato Center (CIP), Nairobi, Kenya

^2^Department of Crops, Horticulture and Soil, Egerton University, Njoro, Kenya

^3^Kenya Agricultural Livestock and Research Organization-Tigoni, Limuru, Kenya

^4^International Potato Center, Hanoi, Vietnam

^5^International Food Policy Research Institute (IFPRI), Washington, USA

*Corresponding author

E-mail: [Evelyne.kihiu@cgiar.org](mailto:Evelyne.kihiu@cgiar.org) (EK)

**S2 Table. DREAMpy scenario estimates.**

**Table A.** Tigoni present value of R&D benefits. Average annual present values in KES million. Baseline scenario

**Table B.** Asante present value of R&D benefits. Average annual present values in KES million. Baseline scenario

**Table C.** Benefits with fungicide application (three sprays per cropping season) with the adoption of 3R-gene Shangi. Average annual present values in KES million

**Table D.** Benefits with a five-year delay in the release of 3R-gene Shangi. Average annual present values in KES million

**Table E.** Benefits with R&D costs doubled. Average annual present values in KES million

**Table F.** Benefits with yield loss averted reduced by a half. Average annual present values in KES million

**Table G.** Benefits of two biotech potato releases: 3R-gene Shangi and new 3R-gene variety. Average annual present values in KES million

**Table H.** Benefits with maximum adoption rates of 3R-gene Shangi. Average annual present values in KES million

**Table I.** Benefits with minimum adoption rates of 3R-gene Shangi. Average annual present values in KES million

**Table A.** Tigoni present value of R&D benefits. Average annual present values in KES million. Baseline scenario

|  | **Region** | $\boldsymbol{\Delta}$**Producer**  **Surplus** | $\boldsymbol{\Delta}$**Consumer**  **Surplus** | $\boldsymbol{\Delta}$**Total**  **Surplus** | $\boldsymbol{\Delta}$**Costs** | $\boldsymbol{\Delta}$**B-**$\boldsymbol{\Delta}$**C** | $\boldsymbol{\Delta}$**B/**$\boldsymbol{\Delta}$**C** | **IRR** |
| --- | --- | --- | --- | --- | --- | --- | --- | --- |
| No spraying | North Rift | -0.01 | 0.01 | 0.00 | 0.03 | **-0.03** | 0.02 | --.-- |
|  | South Rift | -0.01 | 0.01 | 0.00 | 0.09 | **-0.09** | 0.01 | --.-- |
|  | Eastern and Central | 0.17 | 0.05 | 0.23 | 0.31 | **-0.08** | 0.74 | 10 |
|  | **Total** | **0.16** | **0.07** | **0.23** | **0.43** | **-0.20** | **0.54** | **7** |
|  |  |  |  |  |  |  |  |  |
| Triweekly Fungicide Application | North Rift | -0.01 | 0.01 | 0.00 | 0.04 | **-0.05** | -0.01 | --.-- |
|  | South Rift | 0.29 | 0.11 | 0.40 | 0.69 | **-0.29** | 0.58 | 7 |
|  | Eastern and Central | 0.21 | 0.12 | 0.32 | 0.58 | **-0.26** | 0.56 | 7 |
|  | **Total** | **0.49** | **0.23** | **0.72** | **1.32** | **-0.60** | **0.55** | **7** |
|  |  |  |  |  |  |  |  |  |
| Biweekly Fungicide Application | North Rift | -0.01 | 0.01 | 0.00 | 0.07 | **-0.07** | 0.00 | --.-- |
|  | South Rift | 0.56 | 0.20 | 0.77 | 0.91 | **-0.14** | 0.85 | 11 |
|  | Eastern and Central | 0.14 | 0.11 | 0.25 | 0.48 | **-0.23** | 0.52 | 7 |
|  | **Total** | **0.69** | **0.33** | **1.02** | **1.46** | **-0.44** | **0.70** | **9** |
|  |  |  |  |  |  |  |  |  |
| Weekly Fungicide Application | North Rift | 0.00 | 0.00 | 0.00 | 0.01 | **-0.01** | 0.03 | --.-- |
|  | South Rift | 0.02 | 0.01 | 0.03 | 0.04 | **-0.01** | 0.65 | 8 |
|  | Eastern and Central |  |  |  |  |  |  | --.-- |
|  | **Total** | **0.02** | **0.01** | **0.03** | **0.06** | **-0.03** | **0.52** | **6** |
|  |  |  |  |  |  |  |  |  |
|  | **Overall total** | **1.36** | **0.64** | **2.00** | **3.26** | **-1.26** | **0.61** |  |

**Table B.** Asante present value of R&D benefits. Average annual present values in KES million. Baseline scenario

|  | **Region** | $\boldsymbol{\Delta}$**Producer**  **Surplus** | $\boldsymbol{\Delta}$**Consumer**  **Surplus** | $\boldsymbol{\Delta}$**Total**  **Surplus** | $\boldsymbol{\Delta}$**Costs** | $\boldsymbol{\Delta}$**B-**$\boldsymbol{\Delta}$**C** | $\boldsymbol{\Delta}$**B/**$\boldsymbol{\Delta}$**C** | **IRR** |
| --- | --- | --- | --- | --- | --- | --- | --- | --- |
| No spraying | North Rift | -0.01 | 0.01 | 0.00 | 0.01 | **-0.01** | -0.09 | --.-- |
|  | South Rift |  |  |  |  |  |  | --.-- |
|  | Eastern and Central | 1.22 | 0.54 | 1.76 | 0.32 | **1.44** | 5.46 | 29 |
|  | **Total** | **1.21** | **0.55** | **1.76** | **0.33** | **1.43** | **5.35** | **29** |
|  |  |  |  |  |  |  |  |  |
| Triweekly Fungicide Application | North Rift | -0.01 | 0.01 | 0.00 | 0.01 | **-0.01** | -0.09 | --.-- |
|  | South Rift |  |  |  |  |  |  | --.-- |
|  | Eastern and Central | 3.04 | 1.40 | 4.44 | 1.45 | **2.98** | 3.05 | 23 |
|  | **Total** | **3.03** | **1.41** | **4.44** | **1.46** | **2.97** | **3.03** | **23** |
|  |  |  |  |  |  |  |  |  |
| Biweekly Fungicide Application | North Rift | -0.01 | 0.01 | 0.00 | 0.01 | **-0.01** | 0.11 | --.-- |
|  | South Rift |  |  |  |  |  |  | --.-- |
|  | Eastern and Central | 3.01 | 1.37 | 4.39 | 1.45 | **2.93** | 3.02 | 23 |
|  | **Total** | **3.00** | **1.39** | **4.39** | **1.47** | **2.92** | **2.99** | **23** |
|  |  |  |  |  |  |  |  |  |
| Weekly application of fungicides | North Rift | 0.00 | 0.00 | 0.00 | 0.00 | **0.00** | 0.53 | 7 |
|  | South Rift |  |  |  |  |  |  | --.-- |
|  | Eastern and Central |  |  |  |  |  |  | --.-- |
|  | **Total** | **0.00** | **0.00** | **0.00** | **0.00** | **0.00** | **0.53** | **7** |
|  | **Overall total** | **7.24** | **3.35** | **10.59** | **3.26** | **7.33** | **3.25** |  |

**Table C.** Benefits with fungicide application (three sprays per cropping season) with the adoption of 3R-gene Shangi. Average annual present values in KES million

|  | **Region** | $\boldsymbol{\Delta}$**Producer**  **Surplus** | $\boldsymbol{\Delta}$**Consumer**  **Surplus** | $\boldsymbol{\Delta}$**Total**  **Surplus** | $\boldsymbol{\Delta}$**Costs** | $\boldsymbol{\Delta}$**B-**$\boldsymbol{\Delta}$**C** | $\boldsymbol{\Delta}$**B/**$\boldsymbol{\Delta}$**C** | **IRR** |
| --- | --- | --- | --- | --- | --- | --- | --- | --- |
| No spraying | North Rift | 11.1 | 8.8 | 19.8 | 0.1 | 19.7 | 168.0 | 78.0 |
|  | South Rift | 25.2 | 6.9 | 32.1 | 0.1 | 32.0 | 217.2 | 76.2 |
|  | Eastern and Central | 15.2 | 9.8 | 25.0 | 0.1 | 24.9 | 188.2 | 83.0 |
|  | **Total** | **51.5** | **25.4** | **76.9** | **0.4** | **76.5** | **193.0** | **79.2** |
| Triweekly Fungicide Application | North Rift | 4.3 | 6.5 | 10.8 | 0.2 | 10.7 | 66.6 | 62.5 |
|  | South Rift | 88.1 | 25.0 | 113.1 | 0.6 | 112.4 | 186.9 | 74.0 |
|  | Eastern and Central | 21.3 | 26.6 | 47.8 | 0.6 | 47.2 | 80.1 | 68.2 |
|  | **Total** | **113.7** | **58.0** | **171.7** | **1.4** | **170.3** | **125.8** | **70.6** |
| Biweekly Fungicide Application | North Rift | -16.6 | 22.9 | 6.3 | 0.3 | 6.0 | 23.7 | 49.0 |
|  | South Rift | 268.0 | 49.8 | 317.8 | 0.6 | 317.3 | 576.8 | 94.1 |
|  | Eastern and Central | -14.9 | 51.2 | 36.4 | 0.6 | 35.8 | 60.9 | 65.0 |
|  | **Total** | **236.5** | **124.0** | **360.5** | **1.4** | **359.1** | **254.9** | **81.0** |
| Weekly Fungicide Application | North Rift | -0.9 | 3.5 | 2.6 | 0.0 | 2.5 | 58.0 | 61.2 |
|  | South Rift | 14.3 | 3.3 | 17.6 | 0.0 | 17.6 | 437.7 | 89.1 |
|  | Eastern and Central |  |  |  |  |  |  |  |
|  | **Total** | **13.5** | **6.8** | **20.2** | **0.1** | **20.1** | **238.9** | **79.4** |
|  | **Overall total** | 395.7 | 85.0 | 480.7 | 1.3 | 479.3 | 357.6 |  |

**Table D.** Benefits with a five-year delay in the release of 3R-gene Shangi. Average annual present values in KES million

|  | **Region** | $\boldsymbol{\Delta}$**Producer**  **Surplus** | $\boldsymbol{\Delta}$**Consumer**  **Surplus** | $\boldsymbol{\Delta}$**Total**  **Surplus** | $\boldsymbol{\Delta}$**Costs** | $\boldsymbol{\Delta}$**B-**$\boldsymbol{\Delta}$**C** | $\boldsymbol{\Delta}$**B/**$\boldsymbol{\Delta}$**C** | **IRR** |
| --- | --- | --- | --- | --- | --- | --- | --- | --- |
| No spraying | North Rift | 6.8 | 5.7 | 12.5 | 0.1 | 12.4 | 105.7 | 49.2 |
|  | South Rift | 15.3 | 4.5 | 19.8 | 0.1 | 19.6 | 133.7 | 49.3 |
|  | Eastern and Central | 11.6 | 6.4 | 18.0 | 0.1 | 17.9 | 135.7 | 51.9 |
|  | **Total** | **33.7** | **16.5** | **50.3** | **0.4** | **49.9** | **126.1** | **50.2** |
| Triweekly Fungicide Application | North Rift | 3.4 | 6.6 | 9.9 | 0.2 | 9.8 | 61.2 | 44.1 |
|  | South Rift | 90.8 | 25.2 | 116.0 | 0.6 | 115.4 | 191.8 | 52.8 |
|  | Eastern and Central | 20.8 | 26.9 | 47.7 | 0.6 | 47.1 | 79.8 | 46.9 |
|  | **Total** | **115.0** | **58.6** | **173.7** | **1.4** | **172.3** | **127.3** | **49.8** |
| Biweekly Fungicide Application | North Rift | -7.1 | 18.8 | 11.7 | 0.3 | 11.4 | 43.9 | 41.4 |
|  | South Rift | 202.3 | 40.7 | 243.0 | 0.6 | 242.5 | 441.0 | 61.1 |
|  | Eastern and Central | 1.3 | 42.0 | 43.3 | 0.6 | 42.7 | 72.5 | 46.2 |
|  | **Total** | **196.5** | **101.5** | **298.0** | **1.4** | **296.6** | **210.7** | **54.4** |
| Weekly Fungicide Application | North Rift | 0.1 | 3.5 | 3.5 | 0.0 | 3.5 | 79.4 | 46.8 |
|  | South Rift | 13.5 | 3.3 | 16.8 | 0.0 | 16.8 | 416.6 | 60.6 |
|  | Eastern and Central |  |  |  |  |  |  |  |
|  | **Total** | **13.5** | **6.8** | **20.3** | **0.1** | **20.2** | **240.1** | **55.6** |
|  | **Overall total** | **321.9** | **73.7** | **395.6** | **1.3** | **394.3** | **294.4** |  |

**Table E.** Benefits with R&D costs doubled. Average annual present values in KES million

|  | **Region** | $\boldsymbol{\Delta}$**Producer**  **Surplus** | $\boldsymbol{\Delta}$**Consumer**  **Surplus** | $\boldsymbol{\Delta}$**Total**  **Surplus** | $\boldsymbol{\Delta}$**Costs** | $\boldsymbol{\Delta}$**B-**$\boldsymbol{\Delta}$**C** | $\boldsymbol{\Delta}$**B/**$\boldsymbol{\Delta}$**C** | **IRR** |
| --- | --- | --- | --- | --- | --- | --- | --- | --- |
| No spraying | North Rift | 11.1 | 8.8 | 19.8 | 0.2 | **19.6** | 84.0 | 66.0 |
|  | South Rift | 25.2 | 6.9 | 32.1 | 0.3 | **31.8** | 108.6 | 65.0 |
|  | Eastern and Central | 15.2 | 9.8 | 25.0 | 0.3 | **24.7** | 94.1 | 70.6 |
|  | **Total** | **51.5** | **25.4** | **76.9** | **0.8** | **76.2** | **96.5** | **67.3** |
| Triweekly Fungicide Application | North Rift | 5.6 | 10.2 | 15.8 | 0.3 | **15.4** | 48.6 | 57.5 |
|  | South Rift | 148.8 | 39.3 | 188.1 | 1.2 | **186.9** | 155.5 | 70.9 |
|  | Eastern and Central | 23.6 | 41.8 | 65.5 | 1.2 | **64.3** | 54.8 | 71.0 |
|  | **Total** | **178.0** | **91.4** | **269.4** | **2.7** | **266.6** | **98.7** | **66.3** |
| Biweekly Fungicide Application | North Rift | (11.2) | 29.7 | 18.5 | 0.5 | **17.9** | 34.7 | 53.2 |
|  | South Rift | 328.9 | 64.6 | 393.5 | 1.1 | **392.4** | 357.0 | 85.2 |
|  | Eastern and Central | (8.2) | 66.4 | 58.2 | 1.2 | **57.0** | 48.7 | 60.9 |
|  | **Total** | **309.5** | **160.7** | **470.1** | **2.8** | **467.3** | **166.2** | **73.9** |
| Weekly Fungicide Application | North Rift | (0.0) | 5.6 | 5.5 | 0.1 | **5.4** | 62.4 | 62.0 |
|  | South Rift | 21.8 | 5.3 | 27.2 | 0.1 | **27.1** | 336.9 | 84.2 |
|  | Eastern and Central |  |  |  |  |  |  |  |
|  | **Total** | **21.8** | **10.9** | **32.7** | **0.2** | **32.5** | **193.2** | **76.0** |
|  | **Overall total** | 524.8 | 116.1 | 640.9 | 2.7 | **638.2** | 238.4 |  |

**Table F.** Benefits with yield loss averted reduced by a half. Average annual present values in KES million

|  | **Region** | $\boldsymbol{\Delta}$**Producer**  **Surplus** | $\boldsymbol{\Delta}$**Consumer**  **Surplus** | $\boldsymbol{\Delta}$**Total**  **Surplus** | $\boldsymbol{\Delta}$**Costs** | $\boldsymbol{\Delta}$**B-**$\boldsymbol{\Delta}$**C** | $\boldsymbol{\Delta}$**B/**$\boldsymbol{\Delta}$**C** | **IRR** |
| --- | --- | --- | --- | --- | --- | --- | --- | --- |
| No spraying | North Rift | 5.5 | 4.3 | 9.8 | 0.1 | 9.7 | 83.4 | 65.9 |
|  | South Rift | 12.4 | 3.4 | 15.8 | 0.1 | 15.7 | 106.9 | 64.9 |
|  | Eastern and Central | 7.5 | 4.8 | 12.4 | 0.1 | 12.3 | 93.3 | 70.5 |
|  | **Total** | **25.4** | **12.6** | **38.0** | **0.4** | **37.6** | **95.4** | 67.1 |
| Triweekly Fungicide Application | North Rift | 3.6 | 7.2 | 10.9 | 0.2 | 10.7 | 67.0 | 62.7 |
|  | South Rift | 108.6 | 27.8 | 136.5 | 0.6 | 135.9 | 225.6 | 77.2 |
|  | Eastern and Central | 12.9 | 29.6 | 42.5 | 0.6 | 42.0 | 71.2 | 66.5 |
|  | **Total** | **125.2** | **64.7** | **189.9** | **1.4** | **188.5** | **139.2** | **71.8** |
| Biweekly Fungicide Application | North Rift | (3.5) | 20.5 | 17.0 | 0.3 | 16.7 | 64.0 | 62.5 |
|  | South Rift | 217.5 | 44.6 | 262.1 | 0.6 | 261.5 | 475.5 | 90.5 |
|  | Eastern and Central | (1.2) | 45.9 | 44.6 | 0.6 | 44.0 | 74.7 | 67.9 |
|  | **Total** | **212.7** | **111.0** | **323.7** | **1.4** | **322.3** | **228.9** | **79.6** |
| Weekly Fungicide Application | North Rift | (0.0) | 5.6 | 5.5 | 0.0 | 5.5 | 124.8 | 73.8 |
|  | South Rift | 21.8 | 5.3 | 27.2 | 0.0 | 27.1 | 673.7 | 97.2 |
|  | Eastern and Central |  |  |  |  |  |  |  |
|  | **Total** | **21.8** | **10.9** | **32.7** | **0.1** | **32.6** | **386.4** | **88.3** |
|  | **Overall total** | **360.3** | **81.2** | **441.5** | **1.3** | **440.2** | **328.5** |  |

**Table G.** Benefits of two biotech potato releases: 3R-gene Shangi and new 3R-gene variety. Average annual present values in KES million

|  | **Region** | $\boldsymbol{\Delta}$**Producer**  **Surplus** | $\boldsymbol{\Delta}$**Consumer**  **Surplus** | $\boldsymbol{\Delta}$**Total**  **Surplus** | $\boldsymbol{\Delta}$**Costs** | $\boldsymbol{\Delta}$**B-**$\boldsymbol{\Delta}$**C** | $\boldsymbol{\Delta}$**B/**$\boldsymbol{\Delta}$**C** |
| --- | --- | --- | --- | --- | --- | --- | --- |
| No spraying | North Rift | 17.0 | 13.5 | 30.6 | 0.1 | 30.4 | 237.3 |
|  | South Rift | 30.5 | 10.6 | 41.2 | 0.2 | 41.0 | 255.5 |
|  | Eastern and Central | 33.1 | 15.0 | 48.1 | 0.1 | 48.0 | 332.3 |
|  | **Total** | **80.7** | **39.2** | **119.9** | **0.4** | **119.4** | **275.7** |
| Triweekly Fungicide Application | North Rift | 9.7 | 14.8 | 24.6 | 0.2 | 24.4 | 138.7 |
|  | South Rift | 185.4 | 57.2 | 242.6 | 0.7 | 241.9 | 367.8 |
|  | Eastern and Central | 68.0 | 60.5 | 128.5 | 0.7 | 127.9 | 197.2 |
|  | **Total** | **263.1** | **132.6** | **395.7** | **1.5** | **394.2** | **265.9** |
| Biweekly Fungicide Application | North Rift | (11.4) | 41.4 | 30.0 | 0.3 | 29.7 | 103.5 |
|  | South Rift | 420.2 | 90.2 | 510.3 | 0.6 | 509.7 | 849.3 |
|  | Eastern and Central | 27.1 | 92.2 | 119.3 | 0.7 | 118.6 | 183.1 |
|  | **Total** | **435.8** | **223.8** | **659.6** | **1.5** | **658.1** | **427.7** |
| Weekly Fungicide Application | North Rift | 1.3 | 7.5 | 8.9 | 0.0 | 8.8 | 183.3 |
|  | South Rift | 28.1 | 7.2 | 35.3 | 0.0 | 35.2 | 802.6 |
|  | Eastern and Central | - | - | - | - | - | - |
|  | **Total** | **29.4** | **14.7** | **44.1** | **0.1** | **44.1** | **478.4** |
|  | **Overall total** | **809.0** | **410.3** | **1,219.3** | **3.6** | 1,215.7 | **342.7** |

**Table H.** Benefits with maximum adoption rates of 3R-gene Shangi. Average annual present values in KES million

|  | **Region** | $\boldsymbol{\Delta}$**Producer**  **Surplus** | $\boldsymbol{\Delta}$**Consumer**  **Surplus** | $\boldsymbol{\Delta}$**Total**  **Surplus** | $\boldsymbol{\Delta}$**Costs** | $\boldsymbol{\Delta}$**B-**$\boldsymbol{\Delta}$**C** | $\boldsymbol{\Delta}$**B/**$\boldsymbol{\Delta}$**C** |
| --- | --- | --- | --- | --- | --- | --- | --- |
| **No spraying** | North Rift | 2.3 | 6.3 | 8.6 | 0.0 | 8.5 | 193.9 |
|  | South Rift | 22.5 | 6.0 | 28.5 | 0.0 | 28.5 | 708.0 |
|  | Eastern and Central | - | - | - | - | - | - |
|  | **Total** | **24.8** | **12.4** | **37.1** | **0.1** | **37.0** | **438.9** |
| **Tri-Weekly Fungicide Application** | North Rift | 11.2 | 12.4 | 23.6 | 0.2 | 23.5 | 145.5 |
|  | South Rift | 148.5 | 47.7 | 196.2 | 0.6 | 195.6 | 324.4 |
|  | Eastern and Central | 61.6 | 50.8 | 112.5 | 0.6 | 111.9 | 188.2 |
|  | **Total** | **221.4** | **111.0** | **332.3** | **1.4** | **331.0** | **243.5** |
| **Bi-Weekly Fungicide Application** | North Rift | (4.5) | 34.3 | 29.8 | 0.3 | 29.5 | 112.0 |
|  | South Rift | 339.3 | 74.5 | 413.9 | 0.6 | 413.3 | 751.0 |
|  | Eastern and Central | 28.6 | 76.7 | 105.2 | 0.6 | 104.6 | 176.1 |
|  | **Total** | **363.4** | **185.5** | **548.8** | **1.4** | **547.4** | **388.1** |
| **Weekly Fungicide Application** | North Rift | 2.3 | 6.3 | 8.6 | 0.04 | 8.5 | 0.01 |
|  | South Rift | 22.5 | 6.0 | 28.5 | 0.04 | 28.5 | 0.02 |
|  | Eastern and Central | - | - | - | - | - | - |
|  | **Total** | **24.8** | **12.4** | **37.1** | **0.1** | **37.0** | **0.01** |
|  | **Overall Total** | **634.3** | **321.2** | **955.4** | **2.9** | **952.5** | **324.1** |

**Table I.** Benefits with minimum adoption rates of 3R-gene Shangi. Average annual present values in KES million

|  | **Region** | $\boldsymbol{\Delta}$**Producer**  **Surplus** | $\boldsymbol{\Delta}$**Consumer**  **Surplus** | $\boldsymbol{\Delta}$**Total**  **Surplus** | $\boldsymbol{\Delta}$**Costs** | $\boldsymbol{\Delta}$**B-**$\boldsymbol{\Delta}$**C** | $\boldsymbol{\Delta}$**B/**$\boldsymbol{\Delta}$**C** |
| --- | --- | --- | --- | --- | --- | --- | --- |
| **No spraying** | North Rift | 1.9 | 3.6 | 5.6 | 0.1 | 5.4 | 47.1 |
|  | South Rift | 14.9 | 2.9 | 17.8 | 0.1 | 17.6 | 120.4 |
|  | Eastern and Central | 4.0 | 4.0 | 8.1 | 0.1 | 8.0 | 60.9 |
|  | **Total** | **20.9** | **10.5** | **31.4** | **0.4** | **31.1** | **78.9** |
| **Tri-Weekly Fungicide Application** | North Rift | (0.9) | 4.9 | 4.0 | 0.2 | 3.9 | 24.9 |
|  | South Rift | 84.6 | 19.0 | 103.5 | 0.6 | 102.9 | 171.2 |
|  | Eastern and Central | (0.2) | 20.1 | 19.9 | 0.6 | 19.3 | 33.3 |
|  | **Total** | **83.4** | **44.0** | **127.5** | **1.4** | **126.1** | **93.4** |
| **Bi-Weekly Fungicide Application** | North Rift | (11.7) | 15.0 | 3.3 | 0.3 | 3.1 | 12.5 |
|  | South Rift | 182.0 | 32.6 | 214.6 | 0.6 | 214.0 | 389.3 |
|  | Eastern and Central | (17.8) | 33.4 | 15.7 | 0.6 | 15.1 | 26.2 |
|  | **Total** | **152.5** | **81.0** | **233.5** | **1.4** | **232.1** | **165.1** |
| **Weekly Fungicide Application** | North Rift | (1.5) | 2.8 | 1.26 | 0.0 | 1.22 | 0.00 |
|  | South Rift | 12.3 | 2.6 | 14.90 | 0.0 | 14.86 | 0.01 |
|  | Eastern and Central | - | - | - | - | - | - |
|  | **Total** | **10.7** | **5.4** | **16.2** | **0.1** | **16.08** | **0.01** |
|  | **Overall Total** | **267.6** | **141.0** | **408.6** | **3.3** | **405.4** | **125.3** |
